# Tumor Suppressor CADM1 Protects Against Colitis in Inflammatory Bowel Disease Through Enhancing Epithelial Regeneration

**DOI:** 10.3390/ijms27093908

**Published:** 2026-04-28

**Authors:** Yuki Hanaoka-Ikeda, Yumi Tsuboi, Yutaka Kasai, Tomoko Masuda, Hiromi Ichihara, Sumiko Watanabe, Masaru Shinozaki, Yasunori Ohta, Daisuke Matsubara, Yoshinori Murakami

**Affiliations:** 1 Division of Molecular Pathology, Institute of Medical Science, The University of Tokyo, 4-6-1, Shirokanedai, Minato-ku, Tokyo 108-8639, Japany-kasai@nms.ac.jp (Y.K.);; 2 Department of Molecular Biology, Institute for Advanced Medical Sciences, Nippon Medical School, 1-1-5, Sendagi, Bunkyo-ku, Tokyo 113-8602, Japan; 3 Division of Molecular and Developmental Biology, Institute of Medical Science, The University of Tokyo, Tokyo 113-8639, Japan; 4 Department of Surgery, Institute of Medical Science, The University of Tokyo, Tokyo 108-8639, Japan; 5 Department of Laboratory Medicine, Institute of Medical Science, The University of Tokyo, Tokyo 108-8639, Japan; 6 Department of Diagnostic Pathology, Institute of Medicine, University of Tsukuba, Tsukuba 305-8575, Japan

**Keywords:** CADM1, β-catenin, dextran sulfate sodium, epithelial regeneration, human inflammatory bowel disease

## Abstract

Dysregulation of the immune system, gut microbiota alteration, and epithelial dynamics in the colon contribute to the pathogenesis of inflammatory bowel disease (IBD). However, the role of epithelial dynamics, particularly epithelial regeneration, remains incompletely understood. *CADM1* encodes an immunoglobulin-superfamily cell adhesion molecule involved in epithelial adhesion, immune cell interactions, and tumor suppression in colon and various cancers. Here, we investigated the role of *CADM1* in IBD using a murine model of colitis induced by dextran sulfate sodium in both wild-type and conventional *Cadm1*-deficient (*Cadm1*^−/−^) mice. *Cadm1*^−/−^ mice exhibited more severe colitis than wild-type mice with increased mortality (64% vs. 10%) and delayed recovery. *Cadm1*^−/−^ mice showed reduced numbers of Ki-67-positive cells in colonic crypts and delayed epithelial regeneration, whereas no significant differences were observed in epithelial apoptosis, intestinal permeability, or immune responses. Immunohistochemistry revealed that CADM1 expression was restricted to regenerative crypt cells in wild-type mice with nuclear accumulation of β-catenin and phospho-Akt. Furthermore, CADM1 overexpression in colon epithelial cells enhanced Tcf-transcriptional activity in a β-catenin-dependent manner. Immunohistochemistry of human IBD materials revealed that CADM1 expression also correlated with nuclear β-catenin accumulation in crypt epithelial cells. Collectively, CADM1 appears to promote colonic epithelial regeneration through the PI3K/Akt/β-catenin axis to protect against severe epithelial injury in IBD.

## 1. Introduction

Inflammatory bowel disease (IBD), which primarily includes ulcerative colitis (UC) and Crohn’s disease (CD), affects more than 4.9 million individuals worldwide [1]. Multiple mechanisms have been proposed to underlie IBD pathogenesis, including epithelial barrier dysfunction, excessive immune activation, and sustained production of proinflammatory cytokines such as TNF-α, IL-1β, and IL-6 [2,3,4,5]. In addition, accumulating evidence indicates that gut dysbiosis—characterized by reduced microbial diversity and depletion of short-chain fatty acid-producing bacteria—is another key factor in the initiation and perpetuation of IBD. One important mechanism linking dysbiosis to inflammation is mitochondrial dysfunction. Accordingly, epithelial barrier integrity, intestinal permeability, and immune responses are considered major targets of mitochondrial dysfunction in IBD. Current therapeutic strategies for IBD include corticosteroids, immunosuppressants, and biologics targeting tumor necrosis factor-α (TNF-α) or α4β7 integrin [6]. In addition to these approaches, modulators of the microbiome and mitochondrial function, including postbiotics, have emerged as promising therapeutic options [7,8,9]. Furthermore, impaired epithelial repair, in addition to inflammation, has been increasingly recognized as a critical pathological feature of IBD [10,11]. However, the mechanisms underlying defective epithelial regeneration in IBD remain incompletely understood.

Over the past decades, numerous mouse models have been developed to study IBD pathogenesis and evaluate preclinical features. Among them, the dextran sulfate sodium (DSS)-induced colitis model is the most widely used. In this model, various gene-deficient mice are treated with DSS, and the severity of colitis is evaluated to investigate the functional relevance of specific genes. Using this approach, multiple molecules and signaling pathways have been identified as causal or modifying factors in IBD. These include afadin and JAM-A, which regulate epithelial barrier function; IL-21 and IL-23, which act as inflammatory mediators; and Dkk1 and Lgr4, which modulate epithelial regeneration through the inhibition and activation of Wnt/β-catenin signaling, respectively [4,12,13,14,15]. However, the use of constitutive knockout mice limits the interpretation of specific mechanistic pathways, including gut microbiota alteration.

In parallel, genome-wide association studies (GWAS) have identified numerous susceptibility loci for IBD by analyzing genomic DNA from patients and healthy controls. Loci with strong associations (*p* < 1.5 × 10^−8^) include genes involved in immune responses, interactions with intestinal microbiota, epithelial integrity, and related signaling pathways, providing important insights into disease mechanisms [16,17,18]. Among these candidates, cell adhesion molecule 1 (CADM1) is of particular interest because it is expressed in both epithelial and immune cells, although its GWAS *p*-value shows suggestive association (2.3 × 10^−6^) [19]. CADM1 is a member of the immunoglobulin superfamily and functions as a multifunctional cell adhesion molecule [20]. In polarized epithelial cells, CADM1 is localized along the lateral membrane and mediates intercellular adhesion [21]. Notably, CADM1 expression is frequently reduced or lost in various epithelial cancers, including colorectal cancer, through genetic and epigenetic alterations, supporting its role as a tumor suppressor [22]. In addition, CADM1 expression has been observed in regenerating epithelia, such as fetal mouse liver and bile duct epithelium after partial hepatectomy [23], suggesting a potential role in epithelial regeneration.

Beyond its epithelial functions, CADM1 also contributes to immune regulation. It is expressed in natural killer (NK) cells, CD8^+^ T cells, CD103^+^ dendritic cells, and mast cells, where it mediates interactions between immune and epithelial or stromal cells, thereby modulating immune responses and inflammation [21,24]. Thus, CADM1 is positioned at the interface between epithelial integrity and immune regulation. However, despite these characteristics, the role of CADM1 in IBD pathogenesis, particularly in epithelial regeneration, remains unclear.

In the present study, we investigated the role of CADM1 in IBD using a DSS-induced colitis model in *Cadm1*^−/−^ mice and human IBD tissue samples. We found that *Cadm1*^−/−^ mice developed more severe colitis than wild-type controls following DSS treatment. Mechanistically, our data suggest that CADM1 attenuates colitis severity by promoting epithelial regeneration. In wild-type mice, CADM1 expression was upregulated in regenerating crypt epithelium and was associated with PI3K activation and nuclear accumulation of β-catenin. Furthermore, CADM1 enhanced β-catenin-dependent transcriptional activity in a luciferase reporter assay in vitro. Together, this study provides the first evidence that CADM1 protects against colitis, at least in part, through activation of the PI3K/Akt/β-catenin axis, thereby highlighting the importance of epithelial regeneration.

## 2. Results

### 2.1. Cadm1^−/−^ Mice Develop More Severe Colitis than Wild-Type Mice Following DSS Treatment

**Figure 1:**
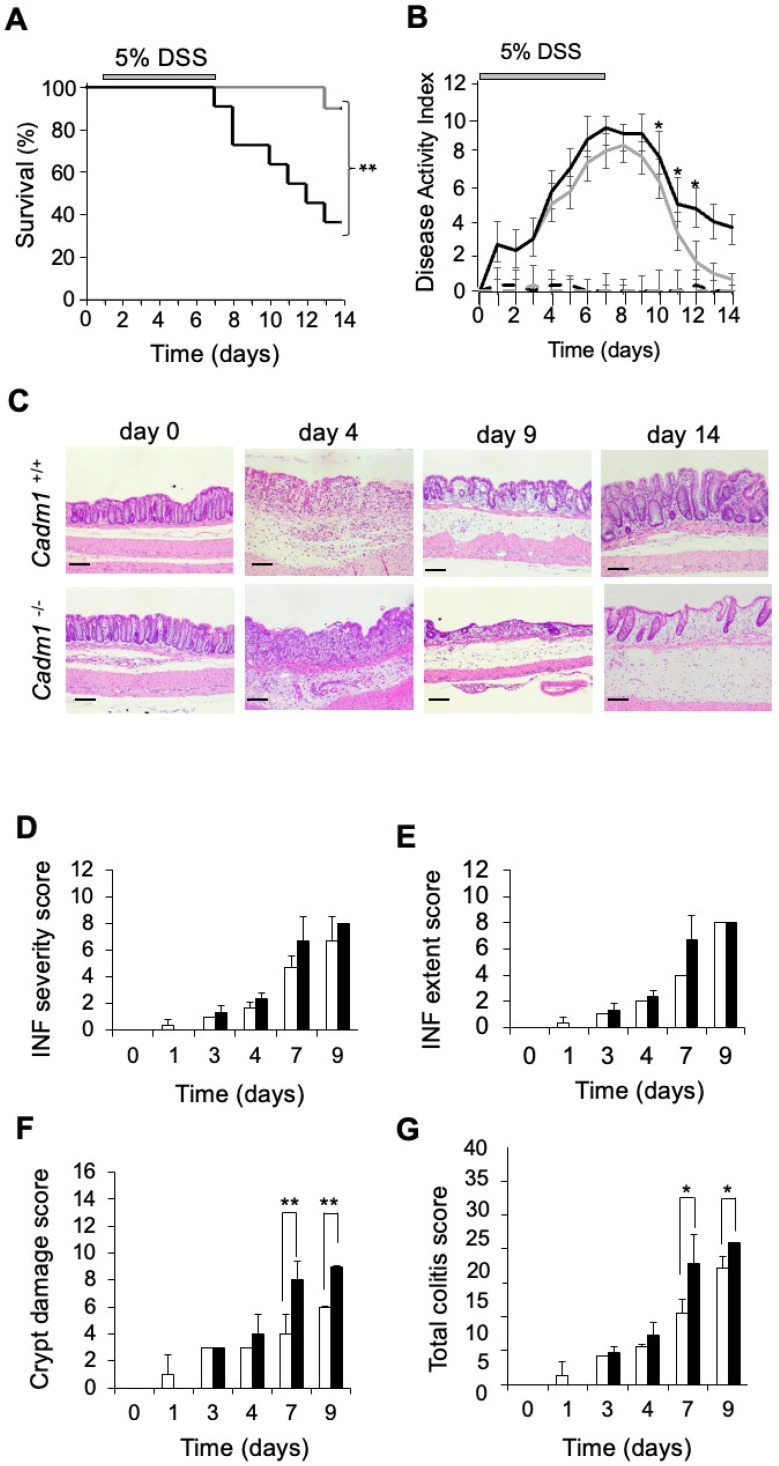
*Cadm1*^−/−^ mice develop colitis with severer phenotype with delayed recovery of intestinal epithelia than wild-type mice after DSS treatment. Mice were exposed to 5% DSS in the drinking water from Day 1 to Day 7, followed by the treatment of water from Day 8 to Day 14. (**A**) Survival rate of DSS-treated *Cadm1*^−/−^ mice (black line, n = 11) and *Cadm1*^+/+^ mice (gray line, n = 10) are 36% and 90%, respectively. ** *p* < 0.01. (**B**) Scores of disease activity index (DAI) were examined in living mice in each time point and represented as means ± standard error (SE) (n = 3 per group). Black and gray solid lines are DSS-treated group of *Cadm1*^−/−^ and *Cadm1*^+/+^ mice, respectively, whereas black and gray dotted lines are water-treated group of *Cadm1*^−/−^ and *Cadm1*^+/+^ mice, respectively. * *p* < 0.05 in post hoc analysis. (**C**) Hematoxylin and eosin staining in large intestinal epithelia of *Cadm1*^+/+^ mice (**upper**) and *Cadm1^−/−^* mice (**lower**) on indicated days. Scale bars, 100 µm (n = 3 per group). (**D**–**G**) Histological scoring of colitis in *Cadm1*^+/+^ mice (open bar) and *Cadm1*^−/−^ mice (closed bar) are shown, including inflammation severity score (0–12) (**D**), inflammation extent score (0–12) (**E**), crypt damage/regeneration score (0–16) (**F**), and total colitis score (Sum of the scores (**D**–**F**): 0–40) (**G**). Data are represented as means ± SD (n = 3 per group). * *p* < 0.05, ** *p* < 0.001 in post hoc analysis.

We previously generated *Cadm1*^−/−^ mice and found that they were born without any apparent abnormalities, except for a defect in spermatogenesis [25]. To investigate the role of CADM1 in IBD, we compared DSS-induced colitis between *Cadm1*^−/−^ and wild-type (*Cadm1*^+/+^) mice. Control groups received DSS-free water for 14 days. In the DSS-treated group, 9 out of 10 (90%) wild-type mice survived, whereas only 4 out of 11 (36%) *Cadm1*^−/−^ mice survived to Day 14 (*p* < 0.01) (Figure 1A). All mice in both genotypes treated with water alone survived. *Cadm1*^−/−^ mice also exhibited greater body weight loss than wild-type mice following DSS treatment (Figure S1). The disease activity index (DAI), calculated as the sum of scores for weight loss (0–4), stool consistency (0–4), and hematochezia (0–4), was significantly higher in *Cadm1*^−/−^ mice than in wild-type mice during the recovery phase at Days 10–12 (*p* < 0.05), although the overall genotype effect did not reach statistical significance (*p* = 0.10) (Figure 1B, Table S2) [26]. Area under the curve (AUC) analysis confirmed a significantly greater cumulative disease burden in *Cadm1*^−/−^ mice compared to wild-type mice (*p* = 0.03). Histological analysis of colon sections further revealed that epithelial regeneration after Day 9 was delayed in *Cadm1*^−/−^ mice compared to wild-type mice (Figure 1C,D). On Day 4, both *Cadm1*^−/−^ and wild-type mice showed comparable levels of erosive changes in the colonic epithelium (Figure 1C). From Day 9 onward, regenerative features, including epithelial and crypt recovery, were evident in wild-type mice. In contrast, *Cadm1*^−/−^ mice showed minimal epithelial and crypt regeneration on Day 9 and even by Day 14, with larger areas of crypt damage than those observed in wild-type mice. However, no significant differences were observed between the two groups in inflammation severity or extent scores (Figure 1D,E). Notably, the crypt damage score was significantly higher in *Cadm1*^−/−^ mice than in wild-type mice on Days 7 (*p* < 0.001) and 9 (*p* < 0.001) (Figure 1F). Consequently, the total colitis score was significantly elevated in *Cadm1*^−/−^ mice compared to wild-type mice on Day 7 (*p* < 0.02) and Day 9 (*p* < 0.05) (Figure 1G). These findings demonstrate that the recovery of intestinal crypts following DSS-induced colitis is markedly delayed in *Cadm1*^−/−^ mice compared to wild-type controls.

### 2.2. CADM1 Expression Is Upregulated in Crypt Epithelial Cells During Recovery from DSS-Induced Colitis

**Figure 2:**
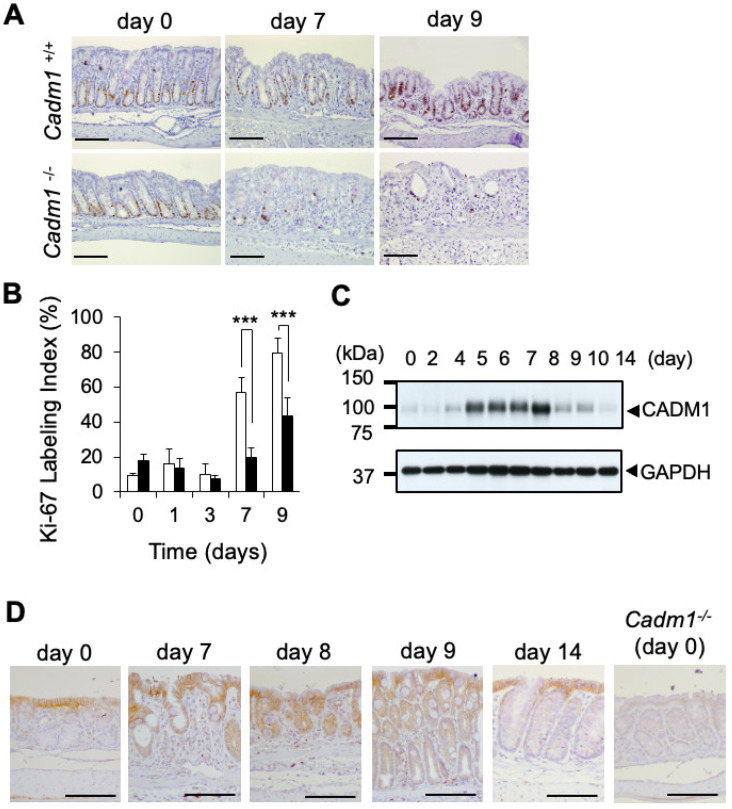
Loss of CADM1 expression exacerbates epithelial proliferative response during recovery from DSS-induced colitis. (**A**) Representative immunohistochemical staining of Ki-67 in the large intestinal epithelia of *Cadm1*^+/+^ mice (**upper**) and *Cadm1*^−/−^ mice (**lower**) on indicated days. Scale bars, 100 µm (n = 3 per group). (**B**) The Ki-67 labeling index of *Cadm1*^+/+^ mice (open bar) and *Cadm1*^−/−^ mice (closed bar), which was defined as the percentage of Ki-67-positive cells among 500 nuclei in the crypts. Data are represented as means ± SD (n = 3 per group). *** *p* < 0.001. (**C**) Western blotting of CADM1 during DSS-treatment. Whole colon lysates of Cadm1^+/+^ mice were prepared on indicated days. (**D**) Representative immunohistochemical staining of CADM1 in crypts of large intestinal epithelia in wild-type mice on indicated days (n = 3 per day). Scale bars, 100 μm.

Since CADM1 is expressed in both epithelial and immune cells, we examined its expression in the context of DSS-induced colitis. CADM1 has been reported to be expressed in dendritic cells and mast cells, both of which may contribute to IBD by activating immune cells such as NK cells and CD8^+^ T cells [21,24]. However, during the colitis-induction phase (Days 0–4), colon tissues from wild-type and *Cadm1*^−/−^ mice showed comparable levels of inflammation, with no significant differences in inflammation severity or extent scores (Figure 2). Next, we analyzed CADM1 expression in murine lamina propria mononuclear cells (LPMCs), which include immune cells, and in epithelial cells, as described in the Supplementary Materials [26,27,28,29,30,31]. RT-PCR analysis of DSS-treated wild-type mice revealed no Cadm1 expression at Day 0, but substantial expression at Days 7, 9, and 14 in intestinal epithelial cells (Figure S2). In contrast, Cadm1 expression was undetectable in LPMCs at all examined time points.

To further explore the role of immune cells, we performed immunohistochemistry on DSS-treated colonic mucosa at Day 8 (Table S1). No co-localization of CADM1 with CD11c (a dendritic cell marker), c-Kit, or Alcian blue staining (mast cell markers) was observed (Figure S3). These findings suggest that CADM1 does not contribute to colitis through expression in dendritic or mast cells in this model. We next investigated whether CADM1 is involved in intestinal epithelial regeneration following DSS treatment. Colonic mucosal homeostasis is regulated by a balance between epithelial cell proliferation and apoptosis. Cell proliferation was assessed using immunohistochemical staining for Ki-67 (Figure 2A). *Cadm1*^−/−^ mice exhibited significantly lower Ki-67 labeling indices (20% on Day 7 and 41% on Day 9) compared to wild-type mice (41% on Day 7 and 79% on Day 9; both *p* < 0.001), demonstrating that Ki-67 expression increased markedly over time in *Cadm1*^+/+^ mice, whereas this increase was attenuated in *Cadm1*^−/−^ mice, resulting in a significant genotype × time interaction (*p* < 0.001) in a two-way repeated measures analysis using a mixed-effects model (Figure 2B).

Apoptosis, assessed by cleaved caspase-3 staining, increased after Day 7 in both genotypes but showed no significant differences between *Cadm1*^−/−^ and wild-type mice (Figure S4). These data indicate that CADM1 promotes epithelial proliferation during regeneration without affecting apoptosis.

We also assessed intestinal permeability, as increased barrier permeability is another mechanism contributing to colitis severity. No statistically significant difference was observed between *Cadm1*^+/+^ and *Cadm1*^−/−^ mice (Figure S5), suggesting that CADM1 is not involved in maintaining epithelial barrier function.

Immunoblotting showed that CADM1 protein expression in the colon of DSS-treated wild-type mice increased from Day 5 to Day 8 and then markedly decreased after Day 9 (Figure 2C). Immunohistochemistry confirmed that, before DSS treatment, CADM1 was restricted to the surface epithelial layer. Between Days 5 and 8, CADM1 expression expanded to crypt epithelial cells (Figure 2D). By Day 9, expression declined in the crypts, and by Day 14 it was absent. These results indicate that CADM1 expression in crypts is transiently induced during epithelial regeneration.

### 2.3. CADM1 Expression Is Associated with Nuclear Accumulation of β-Catenin and Phospho-Akt in Regenerative Crypts Following DSS-Induced Colitis

**Figure 3:**
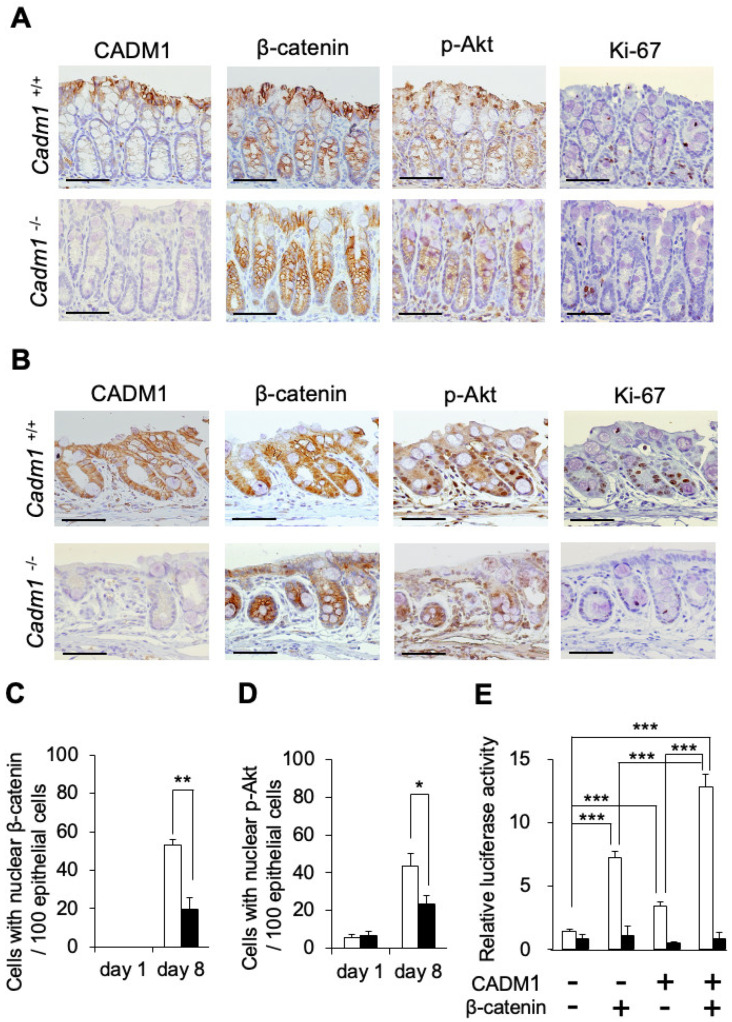
The expression of CADM1 correlates with nuclear accumulation of β-catenin and phospho-Akt in regenerative crypts in DSS-induced colitis. (**A**,**B**) Representative immunohistochemical staining of CADM1, β-catenin, phospho-Akt (pAkt) and Ki-67 in the intestinal epithelia of *Cadm1*^+/+^ mice (**upper**) and *Cadm1*^−/−^ mice (**lower**) on Day 0 (**A**) and Day 8 (**B**) (n = 3 per group). Immunohistochemical staining was performed in serial sections. Scale bars, 50 µm. (**C**,**D**) The number of cells with nuclear accumulated β-catenin (**C**) and pAkt (**D**) per 100 epithelial cells in the crypts from *Cadm1*^+/+^ mice (open bar) and *Cadm1*^−/−^ mice (closed bar) (n = 3 per group) was examined from serial sections of the same crypts. Nuclear positivity was defined as a clearly discernible nuclear staining signal that was distinguishable from background and cytoplasmic staining. Cells showing only cytoplasmic or weak and ambiguous nuclear staining were not considered positive. The results were independently evaluated by two investigators in blinded manner. Data are represented as means ± SD (n = 3 per group). * *p* < 0.05, ** *p* < 0.01. (**E**) CADM1 expression enhances the transcriptional activity of β-catenin in vitro. Colon cancer cells, HCT116, were transiently transfected with the expression vector of β-catenin and/or that of CADM1, together with the luciferase reporter plasmid, pTCF-BP-Luc (open bar), or its mutant plasmid, pTCF-MT-Luc (closed bar), and pRL-TK. Relative luciferase activities are represented as means ± SD (n = 3 per group). *** *p* < 0.001.

β-Catenin plays a pivotal role in intestinal epithelial cell growth and regeneration. Upon activation, both β-catenin and phosphorylated Akt (p-Akt) translocate to the nucleus in proliferating epithelial cells. We assessed the expression and subcellular localization of β-catenin and p-Akt in colonic crypt epithelial cells following DSS treatment using immunohistochemistry. On Day 0, both proteins were localized to the cell membrane in epithelial cells of wild-type and *Cadm1*^−/−^ mice (Figure 3A). On Day 8, β-catenin and p-Akt translocated to the nuclei of crypt epithelial cells in wild-type mice (Figure 3B), coinciding with Ki-67 expression. In contrast, no nuclear accumulation of β-catenin or p-Akt was observed in *Cadm1*^−/−^ mice. The number of cells with nuclear β-catenin and p-Akt was significantly lower in *Cadm1*^−/−^ mice than in wild-type mice on Day 8 (Figure 3C,D). These findings suggest that CADM1 is important for the nuclear translocation of β-catenin and p-Akt during crypt regeneration.

### 2.4. CADM1 Enhances β-Catenin Transcriptional Activity In Vitro

To examine whether CADM1 promotes β-catenin nuclear translocation and activity, we conducted luciferase reporter assays using HCT116 cells transfected with a Tcf-binding site-driven luciferase construct (pTCF-BP-Luc) or a mutant construct (pTCF-MT-Luc) (Figure 3E). Transfection of β-catenin increased luciferase activity, whereas the mutant construct showed only basal activity, confirming Tcf dependency.

Transfection with CADM1 alone also significantly increased luciferase activity. Co-transfection with both β-catenin and CADM1 resulted in a synergistic increase exceeding that of either factor alone, when analyzed using a two-way ANOVA including an interaction term (*p* < 0.0001). These results suggest that CADM1 enhances β-catenin transcriptional activity by promoting its nuclear localization.

### 2.5. CADM1 Expression Correlates with Nuclear Accumulation of β-Catenin in Human IBD Crypts

**Figure 4:**
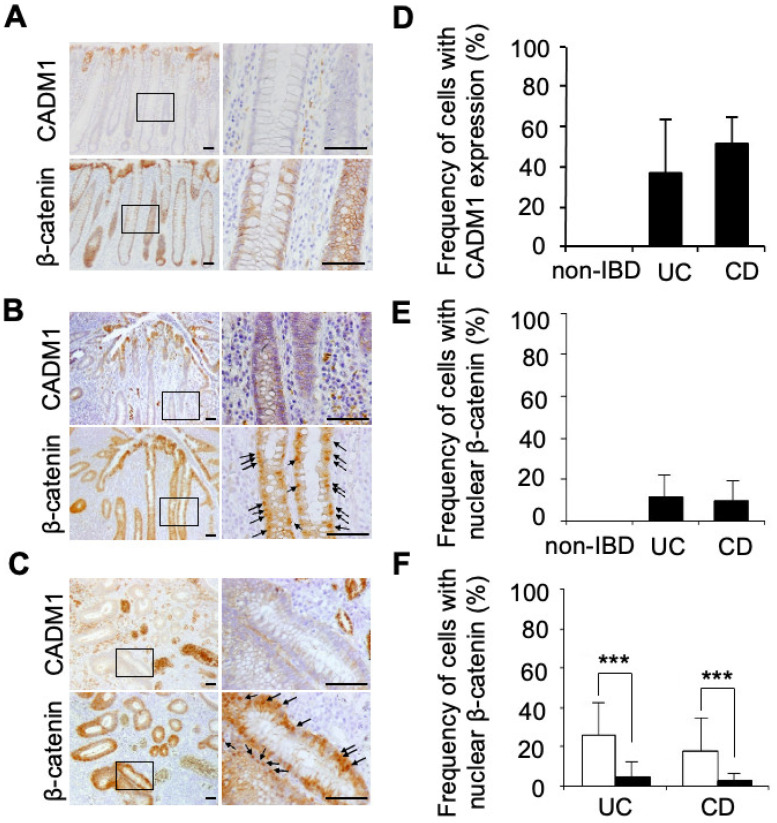
The expression of CADM1 correlates with nuclear accumulation of β-catenin in regenerative crypts from human IBD. (**A**–**C**) Representative immunohistochemical staining of CADM1 and β-catenin in normal colonic mucosa from a non-IBD patient (**A**), human UC tissue (**B**) and human CD tissue (**C**). The right panels are high-power images of the boxed regions in the left panels. Arrows indicate nuclear accumulated β-catenin. Scale bars, 50 µm. (**D**,**E**) The frequency of cells with CADM1 expression (**D**) and with nuclear accumulated β-catenin (**E**) in crypt from human normal, UC and CD tissue. (**F**) The frequency of cells with nuclear accumulated β-catenin in CADM1-positive (open bar) and CADM1-negative (closed bar) crypts in human UC and CD tissue. Student’s *t*-test and a mixed-effects model with patient as a random effect were used to evaluate the statistical difference. *** *p* < 0.001. More than 50 cells were examined in each crypt and 140 crypts for UC tissues from 14 patients and 60 crypts for CD tissues from six patients were counted (**D**–**F**).

Next, we examined human biospecimens after obtaining approval from the institutional ethics committee and informed consent from each participant. We assessed CADM1 and β-catenin expression in colonic crypts from normal tissue, UC, and CD patients using immunohistochemistry. In normal colonic tissue, CADM1 expression was limited to the surface epithelium, and β-catenin was localized to the cell membrane in both surface and crypt epithelial cells (Figure 4A). In contrast, CADM1 was expressed in approximately 40% and 50% of crypt cells in regenerated areas of UC and CD tissues, respectively (Figure 4B–D). Nuclear localization of β-catenin was observed in approximately 10% of regenerative crypt epithelial cells in both UC and CD (Figure 4B,C,E). Among a total of 14,881 crypt epithelial cells from 14 UC patients and 6747 cells from six CD patients, the frequency of nuclear β-catenin was significantly higher in CADM1-positive cells than in CADM1-negative cells (Figure 4F, Table S2). This difference remained statistically significant after accounting for within-patient correlation using a mixed-effects model (UC: *p* < 0.0001; CD: *p* = 0.005), suggesting a strong correlation between CADM1 expression and nuclear β-catenin accumulation.

The proportion of CADM1-positive cells in five mild UC cases, nine moderate/severe UC cases, and six CD cases was 32.8%, 39.4%, and 56.0%, respectively. Meanwhile, the proportion of cells co-expressing CADM1 and nuclear β-catenin was 5.4%, 7.6%, and 8.0%, respectively (Table S5). These data suggest that both the incidence of CADM1 expression and its association with β-catenin nuclear accumulation increase with disease severity in UC and are higher in CD than in UC.

## 3. Discussion

In the present study, we demonstrated that CADM1 plays a critical role in the regeneration of intestinal epithelium following colitis, with possible relevance to the pathogenesis of IBD. To investigate the role of CADM1 in colitis, we employed a DSS-induced colitis model using *Cadm1*^−/−^ and wild-type mice. The results showed that *Cadm1*^−/−^ mice developed more severe colitis than wild-type mice following DSS treatment, with an increased cumulative disease burden (AUC, *p* = 0.03) and a significantly lower survival rate of 36% at day 14, compared to 90% in wild-type mice.

After ruling out the potential involvement of dendritic cells, mast cells, and intestinal permeability (Figures S3 and S5), we focused on the role of CADM1 in epithelial regeneration during IBD. In the recovery phase post-colitis (after day 7), crypt regeneration was notably delayed in *Cadm1*^−/−^ mice, and crypt damage scores were significantly higher than in wild-type mice. These findings suggest that CADM1 contributes importantly to the regeneration of intestinal epithelium following DSS-induced colitis. Furthermore, immunohistochemical analysis of Ki-67 and cleaved caspase-3 revealed that impaired regeneration in *Cadm1*^−/−^ mice was due to reduced epithelial proliferation rather than increased apoptosis (Figure 2 and Figure S4).

CADM1 was originally identified as a tumor suppressor in non-small-cell lung cancer based on its suppressive activity in nude mice [20]. In confluent epithelial cells, CADM1 binds, via its cytoplasmic domain, to spectrin–actin-binding proteins such as 4.1s and PDZ domain-containing scaffold proteins including MAGuKs. siRNA-mediated knockdown of CADM1 has been shown to disrupt epithelia-like structures in polarized HEK293 and Caco-2 cells [32]. Additionally, overexpression of CADM1 suppresses HGF-induced epithelial–mesenchymal transition (EMT) in MDCK cells [27]. Collectively, these findings support a role for CADM1 in the formation and maintenance of epithelial tissue. On the other hand, CADM1 is transiently expressed in fetal mouse liver and in regenerating biliary epithelium in rat liver after partial hepatectomy [23], suggesting a possible role in epithelial regeneration after inflammation.

In the present study, we observed the induction of CADM1 in crypt epithelial cells of inflamed colon tissue, along with phosphorylated Akt and nuclear localization of β-catenin, in association with delayed regeneration (Figure 2D). Furthermore, we demonstrated the co-expression of Ki-67, CADM1, β-catenin, and phospho-Akt in consecutive sections of DSS-treated mouse intestinal epithelium (Figure 3B). These findings suggest that CADM1 promotes intestinal epithelial regeneration through PI3K activation and nuclear accumulation of β-catenin following DSS-induced colitis. β-catenin is widely recognized as a key regulator of epithelial proliferation and regeneration. Indeed, the conditional deletion of β-catenin in intestinal epithelial cells results in rapid crypt loss within 4–5 days after Cre activation [33,34,35]. Wnt/β-catenin signaling plays a central role in epithelial proliferation [36], whereas PI3K/Akt/β-catenin signaling is implicated in epithelial regeneration following colitis [37]. However, the precise molecular mechanisms by which CADM1 activates PI3K and promotes the nuclear localization of β-catenin were not elucidated in this study.

In this context, we previously reported that CADM1 forms a complex with the p85 subunit of PI3K via scaffold proteins such as MPP3 and DLG, thereby activating PI3K and subsequently Akt in Caco-2 cells. This occurs when trans-homophilic interactions of CADM1 are newly established, whereas shRNA-mediated knockdown of CADM1 or treatment with PI3K inhibitors disrupts this complex formation and inactivates PI3K, suggesting a causal role of CADM1 in maintaining PI3K signaling integrity [38]. Together, these data support the hypothesis that CADM1 promotes intestinal epithelial regeneration through the PI3K/Akt/β-catenin signaling pathways. On the other hand, we have not obtained direct evidence linking CADM1 to β-catenin. β-catenin was not identified as a binding partner of CADM1 by mass spectrometry screening [39], and the Wnt/β-catenin pathway was not detected in a chemical inhibitor screen targeting CADM1 signaling [38]. Taken together, these findings suggest that CADM1 promotes nuclear localization of β-catenin indirectly through PI3K/Akt activation.

Consistent with our mouse model findings, human intestinal epithelium from UC and CD patients showed increased numbers of cells with nuclear β-catenin compared to normal controls (Figure 4). Notably, nuclear β-catenin localization was significantly enriched in CADM1-expressing cells in affected tissues (Table S2). These results further support our hypothesis that CADM1 activates PI3K/Akt signaling, thereby promoting nuclear localization of β-catenin. Supporting this, we showed that CADM1 expression in HCT116 cells enhanced TCF-mediated luciferase activity in a β-catenin-dependent manner (Figure 3E), suggesting that the CADM1–PI3K–Akt axis activates β-catenin signaling and may confer protection against DSS-induced colitis.

Conversely, in the absence of CADM1 in *Cadm1*^−/−^ mice, nuclear accumulation of β-catenin is impaired, leading to delayed epithelial regeneration. These findings likely reflect context-dependent functions of CADM1 during epithelial regeneration in vivo. Interestingly, CADM1 expression is induced by all-trans retinoic acid in P19 embryonal carcinoma cells [40], suggesting that CADM1 may act downstream of retinoic acid as a protective factor against colitis, alongside previously reported targets such as CD161^+^ regulatory T cells, intestinal probiotics, and anti-fibrotic pathways [41,42].

Chronic inflammation is a well-known risk factor for colorectal cancer (CRC) in IBD patients, with longer disease duration increasing the risk of IBD-associated CRC [43]. CADM1 has been reported as a tumor suppressor in CRC, where hypermethylation-mediated silencing is frequently observed, particularly in advanced stages of sporadic CRC [22]. Loss of CADM1-mediated adhesion may accelerate CRC progression. Our findings suggest that CADM1 may also function as a tumor suppressor in IBD-associated CRC by promoting epithelial regeneration through PI3K/Akt activation and β-catenin nuclear localization during inflammation recovery. Further studies on the CADM1/PI3K/Akt/β-catenin axis, including therapeutic targeting of CADM1, are warranted.

Recently, Darlington et al. reported that CADM1 promotes proinflammatory signaling in human bowel diseases [44]. They demonstrated that CADM1-expressing myeloid cells contribute to inflammation in human IBD tissues and in chemically induced colitis using a myeloid cell-specific *Cadm1* knockout model. However, they did not address the role of colonic epithelial cells. In contrast, the present study focuses on epithelial CADM1 and demonstrates its role in promoting epithelial regeneration, possibly via PI3K/Akt/β-catenin signaling using a conventional *Cadm1* knockout model. Since CADM1 is expressed in multiple cell types, including immune cells [21], the use of global knockout mice represents a limitation. Nevertheless, our findings highlight a previously underappreciated role of epithelial CADM1 in maintaining mucosal integrity.

Finally, CADM1 may exert contrasting roles in colitis pathogenesis: proinflammatory effects in myeloid cells versus regenerative effects in epithelial cells. We have previously reported similar context-dependent roles in oncogenesis. In epithelial cells, CADM1 maintains structural integrity via cytoskeletal interactions, whereas in lymphocytes it promotes cell motility through Tiam1-mediated RAC activation [45]. These findings underscore the context-dependent functions of CADM1. Although careful interpretation is warranted (see Limitations), our findings provide additional insight into CADM1 as a regulator of cell signaling and epithelial regeneration. Taken together, our results complement those of Darlington et al. and contribute to a more comprehensive understanding of CADM1 in colitis.

### Limitation

Several limitations should be considered in this study. First, this study utilized conventional (global) knockout mice rather than tissue-specific knockout models to assess the function of CADM1 in epithelial cells. Because CADM1 is expressed not only in epithelial cells but also in neuronal and myeloid cells, further studies using epithelial cell-specific Cadm1 knockout mice are required to more precisely define the role of CADM1 in DSS-induced colitis. This is particularly important given that the proinflammatory role of myeloid CADM1 in DSS-induced colitis has been clearly demonstrated by Darlington et al. [44].

Second, in the DSS-induced colitis model, the sample size in most experiments was relatively small (n = 3 per group per time point), which limits statistical power. To address this limitation, we performed two-way repeated-measures ANOVA for DAI scoring (Figure 1B), histological scoring (Figure 1D–G), and the Ki-67 labeling index (Figure 2B) in mice, as well as luciferase assays in vitro (Figure 3E) and analyses of CADM1 expression and β-catenin nuclear localization in human UC and CD samples (Figure 4F), all of which demonstrated significant differences between groups. Nevertheless, these findings should be interpreted with caution due to the limited sample size. In addition, several experimental constraints should be acknowledged, including the exclusive use of male mice (Figure 1, Figure 2 and Figure 3), the lack of quantitative RT-PCR analysis (Figure S2), and the limited time points analyzed for the association between CADM1 expression and β-catenin nuclear localization (only days 0 and 8; Figure 3).

Third, this study does not provide direct mechanistic evidence explaining how CADM1 promotes epithelial regeneration. Therefore, our hypothesis that CADM1 activates the PI3K/Akt/β-catenin pathway to enhance epithelial proliferation should be interpreted cautiously. Further studies are warranted, including rescue experiments with CADM1 overexpression, pharmacological inhibition of the PI3K/Akt/β-catenin axis, and knockdown of key signaling molecules.

Fourth, the human samples from UC and CD patients were limited in number and lacked comprehensive clinical and therapeutic information, and they were not selected based on predefined criteria, which may introduce selection bias. Furthermore, the DSS-induced colitis model has inherent limitations in its relevance to human IBD pathogenesis. This model is primarily driven by chemical epithelial injury and results in acute inflammation largely mediated by innate immune responses, which differs from the complex, chronic, and immune-mediated nature of human IBD. In addition, it does not fully recapitulate the relapsing–remitting disease course or the heterogeneity observed in UC and CD. Therefore, our findings should be interpreted within the context of epithelial injury-driven inflammation. Notably, the inclusion of human UC and CD samples provides partial support for the translational relevance of our findings.

## 4. Materials and Methods

### 4.1. Cells and Antibodies

Human colon cancer HCT116 cells were obtained from the American Type Culture Collection (Manassas, VA, USA) and cultured in McCoy’s 5A medium (Life Technologies, Carlsbad, CA, USA) supplemented with 10% fetal bovine serum (BioWest, Nuaillé, Pays de la Loire, France), 100 units/mL penicillin, and 100 µg/mL streptomycin (Life Technologies). Cells were maintained at 37 °C in a humidified atmosphere containing 5% CO_2_. A rabbit polyclonal anti-CADM1 antibody targeting the C-terminal peptide was used for immunohistochemistry and Western blotting [22]. Other antibodies used are listed in Table S4.

### 4.2. Plasmids

The expression vector for human CADM1, pcTSLC1, and the empty vector pcDNA3.1/Hygro(+) (Life Technologies) were described previously [20]. The reporter plasmids pTCF-BP-Luc, containing seven tandem repeats of the TCF-binding site, and pTCF-MT-Luc, containing mutated TCF-binding sites, as well as an HA-tagged stable β-catenin mutant (pEFBCHA), were also previously described [46].

### 4.3. Animal Experiments

Wild-type C57BL/6 mice were purchased from Japan SLC (Hamamatsu, Japan). *Cadm1*^−/−^ mice were generated as previously described [25] and were backcrossed to the C57BL/6 background for ten generations. For experiments, *Cadm1*^−/−^ mice and their wild-type littermates (7–9 weeks old) derived from intercrosses of *Cadm1*^+/−^ mice were used. All experiments were conducted using male mice to minimize variability associated with sex-dependent differences.

### 4.4. Induction of DSS Colitis

Seven-week-old mice were administered 5% DSS (molecular weight 5000 Da; Fujifilm Wako Pure Chemical, Osaka, Japan) in drinking water from Day 1 to Day 7, followed by DSS-free water from Day 8 to Day 14. Control mice received DSS-free water throughout the experiment. Since a relatively higher concentration of DSS (5%) was used to induce a severe and rapid inflammatory response to C57BL/6 mice in multiple studies, 5% DSS was selected in this study to ensure robust and reproducible induction of colitis, allowing for the clear evaluation of DAI and death rate between *Cadm1*^+/+^ and *Cadm1*^−/−^ mice.

DAI was assessed daily based on weight loss, stool consistency, and occult blood, as described previously (Figure S2) [47]. Mice were sacrificed at the indicated time points. Colons were excised, opened longitudinally, fixed in 10% neutral-buffered formalin, embedded in paraffin, sectioned at 3 µm, and stained with hematoxylin and eosin (H&E). The histological grading of colitis was determined following the criteria of Dieleman et al. [28] (see Table S3). Briefly, inflammation and its depth were graded from 0 to 3, and crypt damage and regeneration were graded from 0 to 4. Crypt damage was scored as: (0) None; (1) Basal 1/3 damaged; (2) Basal 2/3 damaged; (3) Only surface epithelium intact; (4) Entire crypt and epithelium lost. The regeneration score was scored as: (0) Complete regeneration or normal tissue; (1) Almost complete regeneration; (2) Regeneration with crypt depletion; (3) Surface epithelium not intact; (4) No tissue repair. The percentage of affected area was categorized as: (1) 1–25%; (2) 26–50%; (3) 51–75%; (4) 76–100%. Then, the inflammation severity score (0–12), inflammation extent score (0–12), crypt damaging score (0–12), and regeneration score (0–16) were obtained by multiplying the grade and by the percentage involvement, whereas the total colitis score was calculated as the sum of inflammation severity score, inflammation extent score, and crypt damaging score or regeneration score (0–40), as described previously [28]. DAI assessment and histological scoring were independently conducted by two investigators, Y.H.I. and DM, a board-certified pathologist, in a randomized and blinded manner with respect to genotype.

### 4.5. Human Colon Tissue

Human colon tissue was obtained from 14 patients with active UC (median age: 45 years, range: 22–75) and 6 patients with active CD (median age: 30 years, range: 22–36) who underwent surgical resection at the Department of Surgery, Institute of Medical Science, University of Tokyo, between 2009 and 2013. Disease activity in UC was assessed using the Mayo score (range 0–12), comprising stool frequency, rectal bleeding, endoscopic findings, and physician’s global assessment by a board-certified gastroenterologist, MS. CD activity was evaluated using the Crohn’s Disease Activity Index (CDAI), calculated from standard clinical parameters by MS. Among the UC patients, 5 had mild activity (Mayo score < 5), 7 had moderate activity (Mayo score 6–10), and 2 had severe activity (Mayo score > 11). All CD patients had mild activity, with a CDAI of less than 208.

### 4.6. Immunohistochemistry and Evaluation

Paraffin-embedded tissue sections (3 µm thick) were deparaffinized, rehydrated, and subjected to antigen retrieval by boiling in a citrate buffer. Endogenous peroxidase activity was blocked with 3% H_2_O_2_ for 30 min. Sections were then blocked with 2% bovine serum albumin (Sigma-Aldrich, St. Louis, MO, USA) and incubated with the primary antibody at 4 °C overnight. Subsequently, sections were incubated with HRP-conjugated secondary antibodies, developed with a DAB substrate kit (Dako, Glostrup, Denmark), counterstained with hematoxylin, and analyzed using an Olympus BX40 microscope (Olympus, Tokyo, Japan). Immunohistochemical staining for CADM1 and β-catenin was independently evaluated by two investigators, YHI and a board-certified pathologist DM, who were blinded to the clinical data. Staining was semi-quantitatively assessed using an H-score method, which takes into account both the percentage of positive cells and staining intensity. The intensity of staining was graded as 0 (negative), 1 (weak), 2 (moderate), or 3 (strong), and the H-score was calculated as follows:

H-score = (% of cells with intensity 1 × 1) + (% with intensity 2 × 2) + (% with intensity 3 × 3), yielding a total score ranging from 0 to 300. For β-catenin, nuclear localization was specifically evaluated, and only cells with clear nuclear staining were considered positive.

### 4.7. Western Blot Analysis

Mouse tissues were homogenized and lysed on ice for 10 min in a lysis buffer (50 mM Tris-HCl pH 7.5, 150 mM NaCl, 5 mM EDTA, 1% Triton X-100, and protease inhibitors), followed by centrifugation. Protein concentration was determined using a protein assay reagent (Bio-Rad, Hercules, CA, USA). Western blotting was performed as previously described [27].

### 4.8. Assessment of Epithelial Proliferation

Tissue sections were incubated overnight at 4 °C with a monoclonal rabbit anti-Ki-67 antibody (dilution 1:100 in PBS containing 2% BSA). The secondary antibody used was from the LSAB2^®^ System-HRP (Dako). Sections were developed using a DAB substrate kit (Dako, Glostrup, Denmark) and counterstained with hematoxylin. Longitudinal sections were selected to observe the full length of the crypts. The Ki-67 labeling index was calculated as the number of Ki-67-positive cells per 500 epithelial cells [48].

### 4.9. Luciferase Assay

HCT116 cells (4 × 10^4^ cells/well) were seeded in 12-well plates 1 day prior to transfection. Cells were co-transfected using Lipofectamine™ 2000 (Life Technologies) with 0.16 µg of pTCF-BP-Luc or pTCF-MT-Luc, 0.016 µg of pRL-TK (Renilla luciferase control vector; Promega, Madison, WI, USA), 1.36 µg of pcTSLC1 or pcDNA3.1/Hygro(+), and 0.064 µg of pEFBCHA or pBluescript II KS+. After 24 h, cells were lysed, and both Firefly and Renilla luciferase activities were measured using the Dual-Luciferase^®^ Reporter Assay System (Promega) and a Lumat LB9507 luminometer (Berthold Technologies, Bad Wildbad, Germany). Firefly luciferase activity was normalized to Renilla luciferase activity.

### 4.10. Statistical Analyses

Statistical analyses were performed using Student’s *t*-test, two-way ANOVA with interaction terms, and mixed-effects models, as appropriate. For longitudinal or repeated measurements (e.g., DAI, histological scores, and Ki-67 labeling indices), two-way repeated-measures ANOVA or mixed-effects models with subject (mouse or patient) as a random effect were applied. Post hoc multiple comparisons were conducted where appropriate. AUC was calculated for each individual mouse and compared between groups using an unpaired two-tailed Student’s *t*-test. Data are presented as mean ± SD. A *p* value < 0.05 was considered statistically significant. All analyses were performed using SigmaPlot 16 (Systat Software Inc., San Jose, CA, USA) and GraphPad Prism (version 10).

### 4.11. Ethical Considerations

All animal experiments were carried out according to the ARRIVE guidelines with the approval of the Animal Research Committee of the Institute of Medical Science, the University of Tokyo (PA12-09). All studies in which human biospecimens were used were carried out essentially according to the BRISQ guidelines and ethical approval was obtained from the ethics committees of the Institute of Medical Science, the University of Tokyo (IMSUT25-13-0705).

## 5. Conclusions

We demonstrated that CADM1 promotes intestinal epithelial regeneration and exerts a protective role in DSS-induced colitis in mice. This effect is likely mediated, at least in part, through activation of the PI3K/Akt pathway and enhanced nuclear localization of β-catenin leading epithelial proliferation. Loss of CADM1 may therefore contribute to disease pathogenesis in human IBD.

## Data Availability

The data presented in this study are openly available in DMB-IAMS-NMS at https://www.nms.ac.jp/iams/molecular-biology/ accessed on 17 April 2026.

## Supplementary Materials

The following supporting information can be downloaded at: https://www.mdpi.com/article/10.3390/ijms27093908/s1.

## Abbreviations

The following abbreviations are used in this manuscript:

AUC	Area under the curve
BP	Binding promoter
CADM1	Cell adhesion molecule 1
CD	Crohn’s disease
CDAI	Crohn’s Disease Activity Index
CRC	Colorectal cancer
DAB	Diaminobenzidine
DAI,	Disease activity index
Dkk1	Dickkopf-1
DLG	Discs—large
DSS	Dextran sulfate sodium
HA	Hemagglutinin
HEK293	Human embryonal kidney 293
HRP	Horseradish peroxidase
IBD	Inflammatory bowel disease
IL-21	Interleukin-21
IL-23	Interleukin-23
Lgr4	Leucine-rich G protein-coupled receptor-4
LPMC	Lamina propria mucosa cell
MAdCAM-1	Mucosal addressin cell adhesion molecule-1
MAGuK	Membrane-associated guanylate kinase
MDCK	Madin–Darby canine lidney
MPP3	Membrane protein palmitoylated 3
MT	Mutated binding promoter
NK	Natural killer
p-Akt	Phosphorylated Akt
PBS	Phosphate-buffered saline
RL	Renilla luciferase
RT-PCR	Reverse transcription–polymerase chain reaction
SD	Standard deviation
PI3K	Phosphoinositide 3-kinase
Tcf	Transcriptional factor
TK	Thymidine kinase
TNF-α	Tumor necrosis factor-alpha
UC	Ulcerative colitis
Wnt	Wingless

## References

[1] Wang R., Li Z., Liu S., Zhang D. (2023). Global, regional and national burden of inflammatory bowel disease in 204 countries and territories from 1990 to 2019: A systematic analysis based on the global burden of disease study 2019. BMJ Open.

[2] Odenwald M.A., Turner J.R. (2017). The intestinal epithelial barrier: A therapeutic target?. Nat. Rev. Gastroenterol. Hepatol..

[3] Vuyyuru S.K., Kedia S., Sahu P., Ahuja V. (2022). Immune-mediated inflammatory diseases of the gastrointestinal tract: Beyond Crohn’s disease and ulcerative colitis. JGH Open.

[4] Saez A., Herrero-Fernandez B., Gomez-Bris R., Sanchez-Martinez H., Gonzalez-Granado J.M. (2023). Pathophysiology of inflammatory bowel disease: Innate immune system. Int. J. Mol. Sci..

[5] Bai J., Bouwknegt D.G., Weersma R.K., Dijkstra G., van der Sloot K.W.J., Festen E.A.M. (2025). Gene-environmental interactions in inflammatory bowel disease: A systematic review of human epidemiologic studies. J. Crohn’s Colitis.

[6] Lundekvam J.A., Høivik M.L., Anisdahl K., Småstuen M.C., Warren D.J., Bolstad N., Medhus A.W. (2025). Tumour necrosis factor inhibitors in ulcerative colitis: Real-world data on therapeutic drug monitoring and evaluation of current treatment targets (STRIDE II). Ann. Med..

[7] Khatri V., Kalyanasundaram R. (2021). Therapeutic implications of inflammasome in inflammatory bowel disease. FASEB J..

[8] Prajapati S.K., Yadav D., Katiyar S., Jain S., Yadav H. (2025). Postbiotics as Mitochondrial modulators in inflammatory bowel disease: Mechanistic insights and therapeutic potential. Biomolecules.

[9] Kratschmer C., Curiel D.T., Ciorba M.A. (2025). Gut-directed therapeutics in inflammatory bowel disease. Curr. Opin. Gastroenterol..

[10] Shen J., Du S., Zhang Y., Li H., Liu X., Jing J.J. (2026). Bidirectional Crosstalk Between Intestinal Epithelium and Immune Microenvironment in Inflammatory Bowel Disease: Mechanisms and Therapeutic Implications. J. Inflamm. Res..

[11] Li H., Zhang Y., Du S., Shen J., Liu X., Jing J. (2025). “Remodeling the intestinal immune microenvironment”: Immune regulation and tissue regeneration by mesenchymal stem/stromal cells in the repair microenvironment of inflammatory bowel disease. Front. Immunol..

[12] Wang Y., Jiang X., Zhu J., Yue D., Zhang X., Wang X., You Y., Wang B., Xu Y., Lu C. (2016). IL-21/IL-21R signaling suppresses intestinal inflammation induced by DSS through regulation of Th responses in lamina propria in mice. Sci. Rep..

[13] Yin Q., Pi X., Jiang Y., Ren G., Liu Z., Liu H., Wang M., Sun W., Li S., Gao Z. (2021). An immuno-blocking agent targeting IL-1beta and IL-17A reduces the lesion of **DSS**-induced ulcerative colitis in mice. Inflammation.

[14] Yu D., Zhao Y., Wang H., Kong D., Jin W., Hu Y., Qin Y., Zhang B., Li X., Hao J. (2021). IL-1β pre-stimulation enhances the therapeutic effects of endometrial regenerative cells on experimental colitis. Stem Cell Res. Ther..

[15] Liu S., Qian Y., Li L., Wei G., Guan Y., Pan H., Guan X., Zhang L., Lu X., Zhao Y. (2013). Lgr4 gene deficiency increases susceptibility and severity of dextran sodium sulfate-induced inflammatory bowel disease in mice. J. Biol. Chem..

[16] Jarmakiewicz-Czaja S., Zielinska M., Sokal A., Filip R. (2022). Genetic and epigenetic etiology of inflammatory bowel disease: An update. Genes.

[17] Liu J.Z., Van Sommeren S., Huang H., Ng S.C., Alberts R., Takahashi A., Ripke S., Lee J.C., Jostins L., Shah T. (2015). Association analyses identify 38 susceptibility loci for inflammatory bowel disease and highlight shared genetic risk across populations. Nat. Genet..

[18] Cordero R.Y., Cordero J.B., Stiemke A.B., Datta L.W., Buyske S., Kugathasan S., McGovern D.P.B., Brant S.R., Simpson C.L. (2023). Trans-ancestry, Bayesian meta-analysis discovers 20 novel risk loci for inflammatory bowel disease in an African American, East Asian and European cohort. Hum. Mol. Genet..

[19] Barrett J.C., Hansoul S., Nicolae D.L., Cho J.H., Duerr R.H., Rioux J.D., Brant S.R., Silverberg M.S., Taylor M., Barmada M. (2008). Genome-wide association defines more than 30 distinct susceptibility loci for Crohn’s disease. Nat. Genet..

[20] Kuramochi M., Fukuhara H., Nobukuni T., Kanbe T., Maruyama T., Ghosh H.P., Pletcher M., Isomura M., Onizuka M., Kitamura T. (2001). TSLC1 is a tumor-suppressor gene in human non-small-cell lung cancer. Nat. Genet..

[21] Murakami Y., Kasai Y., Masuda T., Ichihara H., Ito T. (2025). Multiple functions of cell adhesion molecule 1 (CADM1) and its role in the pathogenesis of cancer and other diseases. J. Nippon Med. Sch..

[22] Chen K.G., Wang L., Liu P.S., Liu S., Fu X., Zhou Y., Yu H., Li A., Li J., Zhang S. (2010). CADM1/TSLC1 inactivation by promoter hypermethylation is a frequent event in colorectal carcinogenesis and correlates with late stages of the disease. Int. J. Cancer.

[23] Ito A., Nishikawa Y., Ohnuma K., Ohnuma I., Koma Y., Sato A., Enomoto K., Tsujimura T., Yokozaki H. (2007). SgIGSF is a novel biliary-epithelial cell adhesion molecule mediating duct/ductule development. Hepatology.

[24] Takeuchi A., Mel S.B., Miyauchi K., Ishihara C., Onishi R., Guo Z., Sasaki Y., Ike H., Takumi A., Tsuji N.M. (2016). CRTAM determines the CD4+ cytotoxic T lymphocyte lineage. J. Exp. Med..

[25] Yamada D., Yoshida M., Williams Y.N., Fukami T., Kikuchi S., Masuda M., Maruyama T., Ohta T., Nakae D., Maekawa A. (2006). Disruption of spermatogenic cell adhesion and male infertility in mice lacking TSLC1/IGSF4, an immunoglobulin superfamily cell adhesion molecule. Mol. Cell. Biol..

[26] Friedman D.J., Künzli B.M., A-Rahim Y.I., Sevigny J., Berberat P.O., Enjyoji K., Csizmadia E., Friess H., Robson S.C. (2009). CD39 deletion exacerbates experimental murine colitis and human polymorphisms increase susceptibility to inflammatory bowel disease. Proc. Natl. Acad. Sci. USA.

[27] Masuda M., Kikuchi S., Maruyama T., Sakurai-Yageta M., Williams Y.N., Ghosh H.P., Murakami Y. (2005). Tumor suppressor in lung cancer (TSLC)1 suppresses epithelial cell scattering and tubulogenesis. J. Biol. Chem..

[28] Dieleman L.A., Palmen M.J., Akol H., Bloemena E., Peña A.S., Meuwissen S.G., Van Rees E.P. (1998). Chronic experimental colitis induced by dextran sulphate sodium (DSS) is characterized by Th1 and Th2 cytokines. Clin. Exp. Immunol..

[29] Weigmann B., Tubbe I., Seidel D., Nicolaev A., Becker C., Neurath M.F. (2007). Isolation and subsequent analysis of murine lamina propria mononuclear cells from colonic tissue. Nat. Protoc..

[30] Larmonier C.B., Laubitz D., Thurston R.D., Bucknam A.L., Hill F.M., Midura-Kiela M., Ramalingam R., Kiela P.R., Ghishan F.K. (2011). NHE3 modulates the severity of colitis in IL-10-deficient mice. Am. J. Physiol. Gastrointest. Liver Physiol..

[31] Wang L., Srinivasan S., Theiss A.L., Merlin D., Sitaraman S.V. (2007). Interleukin-6 induces keratin expression in intestinal epithelial cells: Potential role of keratin-8 in interleukin-6-induced barrier function alterations. J. Biol. Chem..

[32] Sakurai-Yageta M., Masuda M., Tsuboi Y., Ito A., Murakami Y. (2009). Tumor suppressor CADM1 is involved in epithelial cell structure. Biochem. Biophys. Res. Commun..

[33] Ireland H., Kemp R., Houghton C., Howard L., Clarke A.R., Sansom O.J., Winton D.J. (2004). Inducible Cre-mediated control of gene expression in the murine gastrointestinal tract: Effect of loss of beta-catenin. Gastroenterology.

[34] Fevr T., Robine S., Louvard D., Huelsken J. (2007). Wnt/beta-catenin is essential for intestinal homeostasis and maintenance of intestinal stem cells. Mol. Cell. Biol..

[35] Moparthi L., Koch S. (2019). Wnt signaling in intestinal inflammation. Differentiation.

[36] Nusse R., Clevers H. (2017). Wnt/β-catenin signaling, disease, and emerging therapeutic modalities. Cell.

[37] Lee G., Goretsky T., Managlia E., Dirisina R., Singh A.P., Brown J.B., May R., Yang G.Y., Ragheb J.W., Evers B.M. (2010). Phosphoinositide 3-kinase signaling mediates beta-catenin activation in intestinal epithelial stem and progenitor cells in colitis. Gastroenterology.

[38] Murakami S., Sakurai-Yageta M., Maruyama T., Murakami Y. (2014). Trans-homophilic interaction of CADM1 activates PI3K by forming a complex with MAGuK-family proteins MPP3 and Dlg. PLoS ONE.

[39] Tsuboi Y., Oyama M., Kozuka-Hata H., Ito A., Matsubara D., Murakami Y. (2020). CADM1 suppresses c-Src activation by binding with Cbp on membrane lipid rafts and intervenes colon carcinogenesis. Biochem. Biophys. Res. Commun..

[40] Ito T., Williams-Nate Y., Iwai M., Tsuboi M., Hagiyama M., Ito A., Sakurai-Yageta M., Murakami Y. (2011). Transcriptional regulation of the CADM1 gene by retinoic acid during the neural differentiation of murine embryonal carcinoma P19 cells. Genes Cells.

[41] Abdelhamid L., Luo X.M. (2018). Retinoic acid, leaky gut, and autoimmune diseases. Nutrients.

[42] Povoleri G.A.M., Nova-Lamperti E., Scottà C., Fanelli G., Chen Y.C., Becker P.D., Boardman D., Costantini B., Romano M., Pavlidis P. (2018). Human retinoic acid-regulated CD161+ regulatory T cells support wound repair in intestinal mucosa. Nat. Immunol..

[43] Svrcek M., Nunes P.B., Villanacci V., Beaugerie L., Rogler G., Hertogh G.D., Tripathi M., Feakins R., H-ECCO Group (2018). Clinicopathological and molecular specificities of inflammatory bowel disease-related colorectal neoplastic lesions: The role of inflammation. J. Crohn’s Colitis.

[44] Darlington K.C., Shumway A.J., Sona C., Khaled Z., Steinbach E.C., Kikuchi Y., Fan S., Gartner V., Clough K.M., Nishiyama N.C. (2026). Myeloid Cell Adhesion Molecule 1 (CADM1) Promotes Proinflammatory Signaling in Human Inflammatory Bowel Diseases. Cell. Mol. Gastroenterol. Hepatol..

[45] Masuda M., Maruyama T., Ohta T., Ito A., Hayashi T., Tsukasaki K., Kamihira S., Yamaoka S., Hoshino H., Yoshida T. (2010). CADM1 interacts with Tiam1 and promotes invasive phenotype of human T-cell leukemia virus type I (HTLV-I) transformed cells and adult T-cell leukemia (ATL) cells. J. Biol. Chem..

[46] Ouchi Y., Tabata Y., Arai K., Watanabe S. (2005). Negative regulation of retinal-neurite extension by beta-catenin signaling pathway. J. Cell Sci..

[47] Cooper H.S., Murthy S.N., Shah R.S., Sedergran D.J. (1993). Clinicopathologic study of dextran sulfate sodium experimental murine colitis. Lab. Investig..

[48] Matsuura M., Okazaki K., Nishio A., Matsuura M., Okazaki K., Nishio A. (2005). Therapeutic effects of rectal administration of basic fibroblast growth factor on experimental murine colitis. Gastroenterology.

