# Supplementary material for: Tumor Suppressor CADM1 Protects Against Colitis in Inflammatory Bowel Disease Through Enhancing Epithelial Regeneration
Source: Int J Mol Sci. 2026 Apr 28;27(9):3908. doi: 10.3390/ijms27093908 (PMC13164419; doi:10.3390/ijms27093908)
Supplement: Supplementary file 1 [file ijms-27-03908-s001.zip › ijms-4212031-supplementary.pdf]

List of Supplementary Information

- **Supplementary Methods**
- **Supplementary Table S1**
- **Supplementary Table S2**
- **Supplementary Table S3**
- **Supplementary Table S4**
- **Supplementary Table S5**
- **Supplementary Figure S1**
- **Supplementary Figure S2**
- **Supplementary Figure S3**
- **Supplementary Figure S4**
- **Supplementary Figure S5**

Supplementary Methods

Purification of murine lamina propria mononuclear cells and epithelial cells.

Murine lamina propria mononuclear cells and epithelial cells were purified according to the protocol by Weigmann B. et al (Ref. 1). Briefly, after dissection of the intestinal tissues from mice and digestion of the tissues using collagenase, DNase and dispase, the resulting cells were purified using a series of Percoll gradient to obtain the lamina propria mononuclear cells and epithelial cells.

Ref. 1. Weigmann B.; Tubbe I.; Seidel D.; Nicolaev A.; Becker C.; Neurath M.F. Isolation and subsequent analysis of murine lamina propria mononuclear cells from colonic tissue. *Nat. Protoc.* **2007,** 2, 2307-2311.

RT-PCR analysis

Total RNA was extracted using RNeasy Mini Kit (Qiagen) and first-strand cDNA was synthesized using Transcriptor First Strand cDNA synthesis kit (Roche). The sequences of primers used to detect gene expression were as follows: for *Cadm1*, sense 5′-CTCATTCGGACTATATGCTGTATGTAT-3′ and antisense 5′-CCTCCTTCTGCATTGATTATAGCTGTG-3′; for *Gapdh*, sense 5′-ACCACAGTCCATGCCATCAC-3′ and antisense 5′-TCCACCACCCTGTTGCTGTA-3′.

Immunofluorescence double staining of mouse intestinal epithelia

Immunofluorescence double staining and confocal microscope (Carl Zeiss) were described previously (Ref. 2). Fresh frozen sections of intestinal mucosal tissues at Day 8 after treatment of DSS in C57BL/6 mice and anal epithelial tissues with non-treated C57BL/6 mice were isolated and incubated with primary antibodies at 4°C overnight and anti-chicken IgY and anti-mouse IgG as the secondary antibodies. Anti-CADM1 antibody, 3E1, conjugated with Cy3, anti-CD11c antibody conjugated with FITC, and anti-c-Kit antibody conjugated with Alexa Fluor-488 were used as primary antibodies against CADM1, CD-11c for detecting dendritic cells, and c-Kit for detecting mast cells, respectively. Alcian blue staining was performed in formalin-fixed, paraffin-embedded mouse tissues.

Ref. 2. Masuda M.; Kikuchi S.; Maruyama T.; et al. Tumor suppressor in lung cancer (TSLC)1 suppresses epithelial cell scattering and tubulogenesis. *J. Biol. Chem.* **2005**, 280, 42164-42171.

Assessment of epithelial apoptosis.

After antigen retrieval by boiling in EDTA-containing buffer, tissue sections were incubated with polyclonal rabbit anti-cleaved caspase-3 antibody diluted 1:200 in PBS with 2% BSA at 4 °C overnight. The second antibody used was the HRP-conjugated antibody against rabbit IgG. Sections were developed using a DAB substrate kit and counterstained with hematoxylin. The number of cleaved

caspase-3 positive cells was counted per field, and averages of five fields were calculated as described previously (Ref.3).

Ref. 3. Larmonier C.B.; Laubitz D.; Thurston R.D.; Bucknam A.L.; Hill F.M.; et al. NHE3 modulates the severity of colitis in IL-10-deficient mice. *Am. J. Physiol. Gastrointest. Liver Physiol.* **2011**, 300, G998-1009.

Assessment of intestinal permeability

Intestinal permeability was assessed by enteral administration of FITC-dextran (FD-4, Sigma-Aldrich) as described previously (Ref. 4). FITC-dextran was administered to mice through oral intake of 60 mg/100 g body weight. Four hours later, fluorescence intensity of the serum was measured by FLUO star OPTIMA (BMG LABTECH).

Ref. 4. Wang L.; Srinivasan S.; Theiss A.L.; Merlin D.; Sitaraman S.V. Interleukin-6 induces keratin expression in intestinal epithelial cells: potential role of keratin-8 in interleukin-6-induced barrier function alterations. *J. Biol. Chem.* **2007**, 282, 8219-8227.

Cell proliferation assay

HCT116 cells, SW480 cells from ATCC and LoVo cells from Japanese Cell Research and Resources Bank (JCRB) were transfected with the expression vector of CADM1 or an empty vector. Twenty-four hours after transfection, cells (500 cells/well for HCT116 and LoVo and 1,000 cells/well for SW480) were seeded in a 96-well plate and cultured for indicated days. Cell proliferation rates were measured using Cell Counting Kit-8 (Dojindo). Data are represented as means ± SD (n=3 in each group).

Immunofluorescence analysis of cells

LoVo cells transfected with the expression vector of CADM1 or an empty vector were seeded on poly-Lysine-coated glass bottom dish (Matsunami). Cells were fixed with 4% paraformaldehyde for 20 min, permeabilized with 0.2% Triton X-100 in PBS for 5 min and blocked with 0.2% Triton X-100 and 2% normal goat serum in PBS. Cells were incubated with primary antibody at 4°C overnight and Alexa Fluor-647-conjugated anti-chicken IgY and FITC-conjugated anti-mouse IgG as the secondary antibodies. The coverslips were mounted with Prolong Gold antifade reagent with DAPI (Thermo Fisher Scientific), and the cells were imaged using the Axio Observer D1 epifluorescence microscope (Carl Zeiss).

Table S1. Antibodies used in this study

Antibody	Species	Use	Source	Catalog No.
anti-Ki67	Rabbit monoclonal	Primary Ab	Abcam	ab16667
anti-β-catenin	Mouse monoclonal	Primary Ab	BD Biosciences	#610154
anti-Cleaved Caspase 3	Rabbit polyclonal	Primary Ab	Cell Signaling Technology	#9661
anti-phospho-Akt (pThr308)	Rabbit polyclonal	Primary Ab	Millipore	#07-1398
anti-GAPDH	Mouse monoclonal	Primary Ab	Merck	MAB374-6C5
Anti-SynCAM (TSLC1/CADM1)	Chicken monoclonal	Primary Ab	MBL	CM004-3 3E1
Anti-rabbit IgG, HRP-conjugated	Rabbit polyclonal	Secondary Ab	Millipore	AP132P
Anti-rabbit IgG, HRP-conjugated	Rabbit polyclonal	Secondary Ab	Millipore	AP124P
Alexa Fluor-647-conjugated anti-chicken IgY	Goat polyclonal	Secondary Ab	Abcam	ab150171
FITC-conjugated anti-mouse IgG	Goat polyclonal	Secondary Ab	GeneTex	GTX77238

Table S2. Scoring of disease activity index (DAI*) of mouse colitis.

Score	Weight loss (%)	Stool consistency	Hematochezia**
0	None	Normal	Absence
1	0-10		
2	11-15	Loose stool***	
3	16-20		
4	20<	Diarrhea	Presence

*DAI = Score of weight loss + Score of stool consistency + Sore of hermatochezia

**The presence of gross blood in the stool or anus.

***The formation of a stool that readily becomes paste on anus of mice.

Ref. 5. Friedman D.J.; Künzli B.M.; A-Rahim Y.I.; Sevigny J.; Berberat P.O.; Enjyoji K.; Csizmadia E.; Friess H.; Robson S.C. CD39 deletion exacerbates experimental murine colitis and human polymorphisms increase susceptibility to inflammatory bowel disease. *Proc Natl Acad Sci U S A* **2009**, 106, 16788-16793.

Table S3. Histological grading of mouse colitis.

Features graded	Grade	Description
Inflammation	0	None
	1	Slight
	2	Moderate
	3	Severe
Extent	0	Non
	1	Mucosa
	2	Mucosa and submucosa
	3	Tranmural
Regeneration	4	No tissue repair
	3	Surface epithelium not intact
	2	Regeneration with crypt depletion
	1	Almost complete regeneration
	0	Complete regeneration or normal tissue
Crypt damage	0	None
	1	Basal 1/3 damaged
	2	Basal 2/3 damaged
	3	Only surface epithelium intact
	4	Entire crypt and epithelium lost
Percent involvement	1	1–25%
	2	26–50%
	3	51–75%
	4	76–100%

Inflammation and its depth were graded from 0 to 3, and crypt damage or regeneration was graded from 0 to 4. The percentage of affected area was categorized as: (1) 1–25%; (2) 26–50%; (3) 51–75%; (4) 76–100%. Then, inflammation severity score (0-12), inflammation extent score (0-12), crypt damaging score (0-1), and regeneration score (0-16) were obtained by multiplying the grade and by the percentage involvement, whereas the total colitis score was calculated as the sum of inflammation severity score, inflammation extent score, and crypt damaging score or regeneration score (0-40), as described in Ref. 6.

Ref.6. Dieleman L.A.; Palmen M.J.; Akol H.; et al. Chronic experimental colitis induced by dextran sulphate sodium (DSS) is characterized by Th1 and Th2 cytokines. *Clin. Exp. Immunol.* **1998**, 114, 385-391.

Table S4. Correlation of CADM1 expression and nuclear localization of β-catenin in colon tissues from UC and CD patients.

	β-catenin localization		
	Nuclear	Diffuse cytoplasmic	χ^2 *(p)*
A. Ulcerative colitis*			
CADM1 (+)	1329	5644	872
CADM1(-)	307	7601	(0.000)
B. Crohn's disease**			
CADM1 (+)	581	3199	327
CADM1(-)	68	2899	(0.000)

* 140 fields from 14 cases (total 14,881 crypt epithelial cells) were examined.

** 60 fields from 6 cases (total 6,747 crypt epithelial cells) were examined.

Table S5. CADM1 expression, co-incidental nuclear β-catenin in intestinal epithelia and disease activities of UC and CD patients.

	No. of cases examined	CADM1 expression (%)	nuclear β-catenin (%)	CADM1(+) with nuclear β-catenin (%)
A. Ulcerative colitis*				
Mild	5	32.8	7.4	5.4
Moderate + Severe	9	39.4	9,4	7.6
Total	14	37.1	8.7	6.8
B. Crohn's disease**				
Mild	6	56.0	9.0	8.0
Total	6	56.0	9.0	8.0

* 140 fields from 14 cases (10 fields per each case) were examined.

** 60 fields from 6 cases (10 fields per each case) were examined.

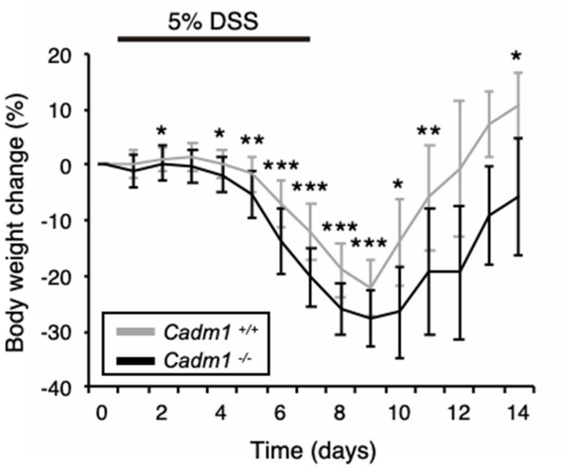

Figure S1. *Cadm1*$^{-/-}$ mice develop colitis with severer phenotype than *Cadm1*$^{+/+}$ mice after DSS-treatment. Percent body weight change of mice treated with DSS in *Cadm1*$^{-/-}$ mice (black line) and *Cadm1*$^{+/+}$ mice (gray line) were examined in living mice in each time point and represented as means ± standard deviation (SD) (n=3 in each group). *$P < 0.05$, **$P < 0.01$, ***$P < 0.001$.

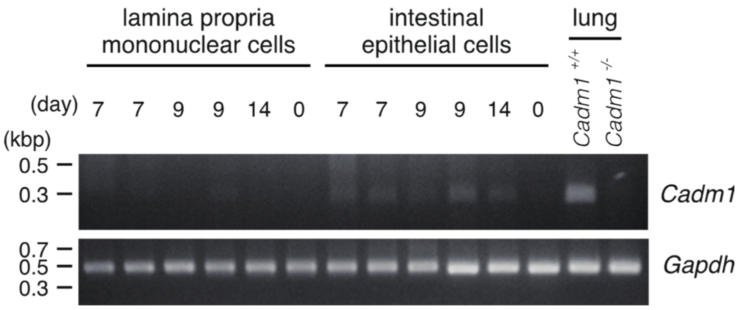

Figure S2. CADM1 is expressed in intestinal epithelial cells during recovery from DSS-induced colitis. RT-PCR of *Cadm1* was performed using lamina propria mononuclear cells and intestinal epithelial cells prepared from DSS-treated *Cadm1*$^{+/+}$ mice on indicated days.

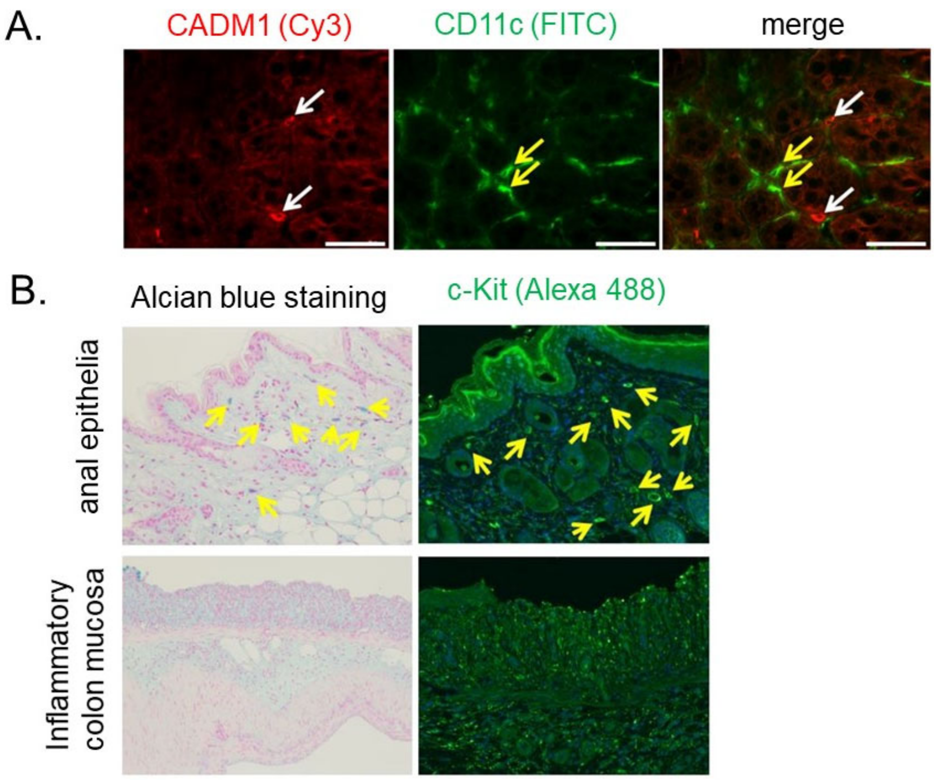

Figure S3. Immunofluorescence staining of CADM1 expressing cells, dendritic cells, and mast cells in intestinal mucosa from DSS-induced colitis in C57BL/6 mice. Intestinal mucosa of wild-type mice at Day 8 after DSS treatment were analyzed. (A) Staining of CADM1-expressing cells (anti-CADM1 antibody-Cy3) and dendritic cells (anti-CD11c-FITC). (B) Staining of mast cells with anti-c-Kit-Alexa Fluor 488 (right) and with Alucian blue (left).

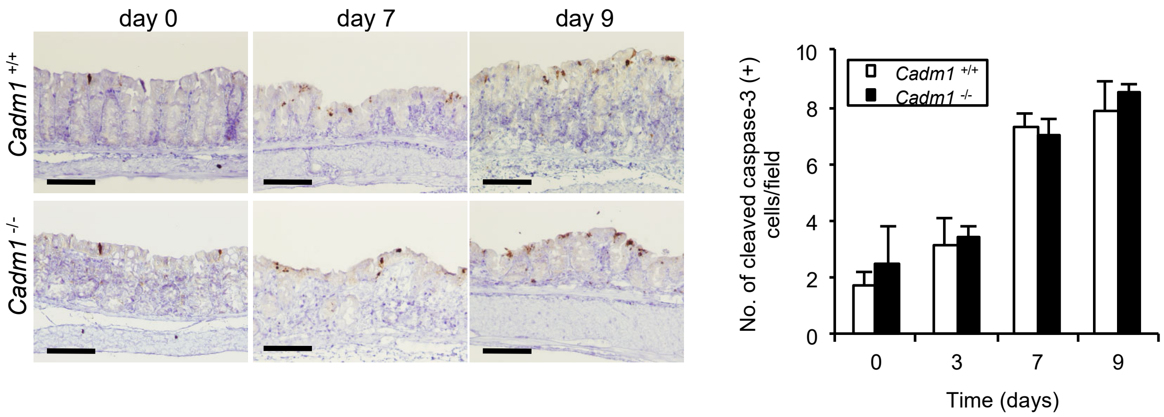

Figure S4. Loss of CADM1 expression does not affect apoptosis of intestinal epithelia during recovery from DSS-induced colitis. (A) Representative immunohistochemical staining of cleaved caspase-3 in the intestinal epithelia of *Cadm1*$^{+/+}$ mice (upper) and *Cadm1*$^{-/-}$ mice (lower) on indicated days. Scale bars, 100 μm. (B) Staining in *Cadm1*$^{+/+}$ mice (open bar) and *Cadm1*$^{-/-}$ mice (closed bar) was scored by counting the number of cleaved caspase-3-positive cells per high-power field of vision (x200). Averages of 5 fields in 3 randomly chosen sections were analyzed for each mouse. Data are represented as means ± SD (n=3 in each group).

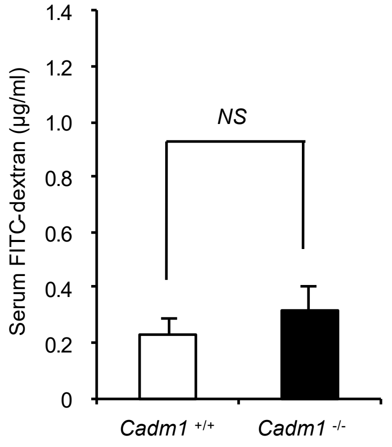

Figure S5. CADM1 does not affect barrier function of intestinal epithelia. Intestinal permeability was determined from serum FITC-dextran concentration at 4 hours after administration. *Cadm1*$^{+/+}$ mice (open bar) and *Cadm1*$^{-/-}$ mice (closed bar) without DSS treatment were examined. Data are represented as means ± SD (n=3 in each group).
